# Mapping the Clinical Competencies Required for Nurses to Use Point-of-Care Ultrasound: A Scoping Review

**DOI:** 10.3390/healthcare14142196

**Published:** 2026-07-20

**Authors:** João Henrique Ribeiro Machado Silva, Jennifer Midiani Gonella, Marília Tomé Maia, Priscilla Roberta Silva Rocha, Fernanda Raphael Escobar Gimenes

**Affiliations:** 1 Ribeirão Preto College of Nursing (EERP), University of São Paulo (USP), Ribeirão Preto 14040-902, Brazil; jennifermidiani@gmail.com (J.M.G.); mariliatomemaia@gmail.com (M.T.M.); fregimenes@eerp.usp.br (F.R.E.G.); 2 Faculty of Health Sciences and Technology (FCTS), University of Brasília (UnB), Brasília 72220-275, Brazil; priscillarocha@unb.br

**Keywords:** ultrasonography, clinical competence, professional performance evaluation, continuing education, nursing

## Abstract

**Highlights:**

**What are the main findings?**

- Clinical competencies required for nurses to use point-of-care ultrasound (PoCUS) were identified across all four levels of Miller’s Pyramid; however, competency development was uneven and remained predominantly focused on technical and procedural performance.
- Competencies related to clinical reasoning, interpretive judgment, and autonomous decision-making were inconsistently described and rarely supported by structured assessment strategies.

**What are the implications of the main findings?**

- Nursing-specific clinical competency frameworks are needed to align PoCUS education, assessment, and scope of practice with safe, ethical, and clinically meaningful nursing practice.
- Longitudinal competency-based education is essential to support progression from foundational knowledge to autonomous clinical practice.

**Abstract:**

**Background/Objectives**: Point-of-care ultrasound (PoCUS) has been increasingly incorporated into nursing practice across diverse clinical settings. However, the absence of a clear, nursing-specific conceptualization of the clinical competencies required for its use continues to limit its safe and effective integration. This scoping review aimed to map and synthesize the clinical competencies required for nurses to use PoCUS and to examine how these competencies have been described and assessed in the literature. **Methods**: A scoping review was conducted in accordance with the JBI Collaboration methodology and reported according to the PRISMA-ScR guideline. The protocol was registered in the Open Science Framework. Searches were conducted on 9 April 2025 in PubMed/MEDLINE, Embase, CINAHL, Web of Science, and the Cochrane Library, and were complemented by gray literature searches in Google Scholar and ProQuest Dissertations & Theses Global. Data synthesis was guided by Miller’s Pyramid of clinical competence. **Results**: A total of 2481 records were identified. After removal of 540 duplicates, 1941 records were screened. Of these, 128 full-text articles were assessed for eligibility and 28 studies were included in the final synthesis. Technical and procedural competencies predominated, particularly those related to image acquisition and ultrasound-guided interventions. By contrast, competencies associated with clinical reasoning, interpretive judgment, and autonomous decision-making were described inconsistently. **Conclusions**: Although PoCUS use by nurses is expanding, clinical competence development remains uneven and predominantly focused on technical proficiency. Nursing-specific frameworks, competency-based education, and validated assessment tools are essential to ensure the safe, ethical, and effective integration of PoCUS into clinical practice.

## 1. Introduction

Point-of-care ultrasonography (PoCUS) has emerged as a promising technology that complements clinical assessment, enables real-time visualization, and contributes substantially to clinical reasoning, diagnostic accuracy, and the performance of bedside interventions across diverse healthcare settings [1]. Its use has progressively expanded beyond medical practice, and evidence indicates that, when appropriately trained, nurses can perform ultrasound scans accurately, interpret images with clinical relevance, and use this tool to enhance care delivery, with positive implications for patient safety and quality of care [2,3]. In this context, PoCUS has potential for incorporation into the Nursing Process, as it may support the identification of patient needs, the prioritization of problems, selection of interventions, and the evaluation of outcomes sensitive to nursing care [4]. Furthermore, its use strengthens clinical decision-making and facilitates effective communication within multidisciplinary healthcare teams [5].

Within nursing practice, PoCUS has been applied across different patient populations and levels of care, contributing to early recognition of physiological alterations, complementing physical examination, enhancing clinical reasoning, and supporting monitoring of clinical progression [3,6,7]. Its application also assists in identifying defining characteristics and risk factors related to nursing diagnoses, as well as in guiding invasive procedures, particularly in emergency, critical care, and procedural contexts [8,9]. Thus, the integration of PoCUS into nursing practice is closely aligned with nurses’ ethical and professional responsibility to reduce healthcare-associated harm and optimize care outcomes [8].

Internationally, the implementation and regulation of nurse-performed PoCUS vary across healthcare systems, reflecting differences in professional scope of practice, educational requirements, and regulatory frameworks. In Brazil, the use of bedside ultrasonography by nurses was formally recognized by the Federal Council of Nursing (Conselho Federal de Enfermagem—COFEN) through Technical Chamber Opinion No. 0052/2021/CTLN/DGEP/COFEN [10]. This document establishes that nurses are not permitted to issue diagnostic reports and must not use PoCUS for nosological diagnosis, in accordance with the legal restriction set forth in item VII of Article 4 of Law No. 12,842/2013 [10]. It further emphasizes that this practice requires specific training and demonstrated technical competence to ensure safe, qualified, and ethically and legally compliant professional practice [10].

Despite the increasing use of point-of-care ultrasonography (PoCUS) across healthcare settings, its consolidation as a safe, effective, and professionally grounded nursing practice remains uneven. A major challenge is the absence of a clear, nursing-specific conceptualization of the clinical competence required for PoCUS use [11,12]. Rather than being limited to technical proficiency, competence in nursing PoCUS should be understood as a multidimensional construct integrating theoretical knowledge, psychomotor skill, image interpretation, clinical reasoning, situational awareness, ethical judgment, and safe decision-making in real care environments [13]. However, the literature remains fragmented and methodologically heterogeneous, with substantial inconsistencies in how these competencies are defined, operationalized, taught, and assessed.

Competency-based education frameworks provide an important lens through which this gap can be addressed. Among these, Miller’s Pyramid [14] provides a particularly valuable model for understanding the progression of clinical competence from knowledge acquisition (“knows”) and knowledge application (“knows how”) to performance in controlled settings (“shows how”) and autonomous practice in real-world care (“does”) [14,15]. In the context of nursing PoCUS, this framework is especially relevant because it conceptualizes competence not as the isolated mastery of technical tasks, but as a continuum of professional development culminating in safe, context-sensitive, and clinically meaningful practice.

Although Miller’s Pyramid offers a useful structure for organizing competencies according to progressive levels of performance, it may not fully capture the complexity of nursing practice. Nursing competence extends beyond observable technical performance and includes relational, ethical, contextual, and interprofessional dimensions that are not explicitly represented within the hierarchical structure of the model. Therefore, the pyramid should be interpreted as an analytical framework rather than as a comprehensive representation of nursing competence. Although widely adopted in health professions education, its application to PoCUS in nursing has not yet been systematically examined or synthesized. Identifying how existing studies explicitly or implicitly align PoCUS-related competencies with these hierarchical levels is essential to reveal weaknesses in current training approaches, limitations in assessment strategies, and opportunities to develop nursing-specific evaluative instruments.

Against this background, a scoping review is warranted to systematically map the clinical competencies required for nurses to use PoCUS, examine how these competencies have been conceptualized and assessed in the literature, and analyze their alignment with established competency-based frameworks. By synthesizing the available evidence through the lens of Miller’s Pyramid [14,15], this review seeks to move beyond a descriptive account of educational experiences and training initiatives and provide a structured, theory-informed understanding of competence development in nursing PoCUS. This approach is essential to support the design of educational programs, the development of valid and contextually appropriate assessment instruments, and the advancement of regulatory and professional guidance capable of sustaining the safe, ethical, and effective integration of PoCUS into nursing practice.

### Aim

This scoping review aimed to systematically map and synthesize the clinical competencies required for nurses to use PoCUS. This includes both explicitly stated competencies and those inferred from educational outcomes, procedural performance, assessment activities, and descriptions of clinical practice. Using Miller’s Pyramid [14] of clinical competence as an analytical framework, the review sought to identify patterns, gaps, and inconsistencies in the progression from foundational knowledge to autonomous clinical practice, thereby generating theory-informed evidence to support the development of nursing-specific education, assessment instruments, and competency-based PoCUS training models.

## 2. Materials and Methods

### 2.1. Design

This scoping review was conducted in accordance with the JBI methodology [16] and is reported according to the Preferred Reporting Items for Systematic Reviews and Meta-Analysis Protocols—extension for Scoping Reviews (PRISMA-ScR) [17] guidelines. The review protocol was registered in the Open Science Framework (OSF) [18] and is available at https://doi.org/10.17605/OSF.IO/XKFDH.

### 2.2. Search Strategy

A comprehensive, systematic search strategy was developed in collaboration with two experienced health sciences librarians. Searches were conducted on 9 April 2025 across the following electronic databases: PubMed (via the National Institutes of Health), Embase (via Elsevier), CINAHL (via EBSCOhost), Web of Science Core Collection (via Clarivate), and the Cochrane Library. To enhance coverage and reduce publication bias, gray literature was also searched in Google Scholar and ProQuest Dissertations & Theses Global.

Controlled vocabulary terms (MeSH and DeCS) and relevant keywords related to nursing, point-of-care ultrasound, and clinical competence were combined using Boolean operators. No restrictions were applied regarding language or year of publication. When necessary, articles were translated for eligibility assessment. The complete search strategies for each database are presented in Appendix A.

### 2.3. Inclusion and/or Exclusion Criteria

The review question was formulated using the Population–Concept–Context (PCC) framework recommended by JBI [16]: P (nurses), C (clinical competencies required for PoCUS use), and C (healthcare settings in which nurses perform PoCUS). Based on this framework, the guiding research question was: What clinical competencies are required for nurses to use point-of-care ultrasound in healthcare settings?

This review included primary studies, review articles, clinical or educational guidelines, and other publications addressing clinical competencies, skills, or competency assessment related to PoCUS use by nurses. Studies were excluded if they (1) focused exclusively on physicians or other health professionals without explicitly including nurses; (2) addressed ultrasound use unrelated to point-of-care or bedside applications; or (3) discussed educational interventions without reference to competency development or assessment.

### 2.4. Search Outcome

The search conducted on 9 April 2025 yielded 2293 records. Of these, 234 duplicates were identified and automatically removed using EndNote (https://www.myendnoteweb.com/EndNoteWeb.html acessed on 9 April 2025), leaving 2059 records. These records were then exported to Rayyan (https://www.rayyan.ai/ acessed on 15 April 2025) and combined with 188 records identified through gray literature sources, resulting in 2247 records. A second automated duplicate-detection process was conducted in Rayyan, leading to the removal of an additional 306 duplicates. Consequently,1941 records remained for title and abstract screening.

During the initial screening, 1813 records that did not meet the predefined eligibility criteria were excluded. The full texts of the remaining 128 potentially relevant reports were independently assessed by two reviewers. Of these, 100 reports were excluded because they did not meet the eligibility criteria established according to the Population, Concept, and Context framework. framework. Disagreements at any stage of the selection process were resolved through discussion and consensus. When consensus could not be reached, a third reviewer was consulted.

### 2.5. Quality Appraisal

Following JBI recommendations for scoping reviews [16], no formal quality appraisal was undertaken, as the purpose of this review was to map available evidence rather than evaluate study quality.

### 2.6. Data Abstraction

Data were charted independently by two reviewers using a standardized extraction form developed in Microsoft Excel and informed by the JBI [16] data extraction template for scoping reviews. Before formal data extraction, the charting form was pilot-tested on a small sample of included studies and refined by the review team to improve consistency and clarity. Charted data were compared between reviewers, and discrepancies were resolved through discussion and consensus. When necessary, a third reviewer was consulted. No contact with study authors was required for clarification of the extracted information.

Extracted data included: publication characteristics (year, country, and language), study design and clinical setting, nursing role and level of training, PoCUS application and clinical context, description of clinical competencies, and educational strategies and assessment approaches. In addition, competencies described in each study were mapped according to the four hierarchical levels of Miller’s Pyramid of clinical competence: knows, knows how, shows how, and does [14].

Competencies were classified as involving clinical reasoning when studies explicitly described the interpretation of ultrasound findings, integration of PoCUS results with patient assessment, recognition of uncertainty, prioritization of clinical problems, or clinical decision-making informed by ultrasound findings. This operational definition was applied consistently during data charting and competency mapping according to Miller’s Pyramid [14]. When a competency aligned with multiple levels of Miller’s Pyramid, classification was based on the highest level of performance explicitly described in the study. Any discrepancies or ambiguous cases were discussed between the reviewers and resolved through consensus, with a third reviewer consulted when necessary.

Consistent with JBI guidance for scoping reviews [16], no formal methodological quality appraisal was undertaken, as the objective of this review was to map the scope, nature, and conceptualization of existing evidence rather than to assess intervention effectiveness or risk of bias.

### 2.7. Synthesis

The synthesis followed a descriptive and analytical approach. Extracted data were summarized narratively and organized thematically according to the review objectives. Clinical competencies were analyzed and grouped using Miller’s Pyramid [14] as an analytical framework, enabling examination of how competencies were distributed across different levels of clinical performance and professional autonomy.

Tables and figures were developed to illustrate the geographical distribution of studies, methodological characteristics, and the alignment of the identified competencies with the hierarchical levels of Miller’s model [14]. This approach enabled identification of patterns, gaps, and inconsistencies in the conceptualization and assessment of nursing competencies for PoCUS use.

## 3. Results

### 3.1. Study Selection

**Figure 1:**
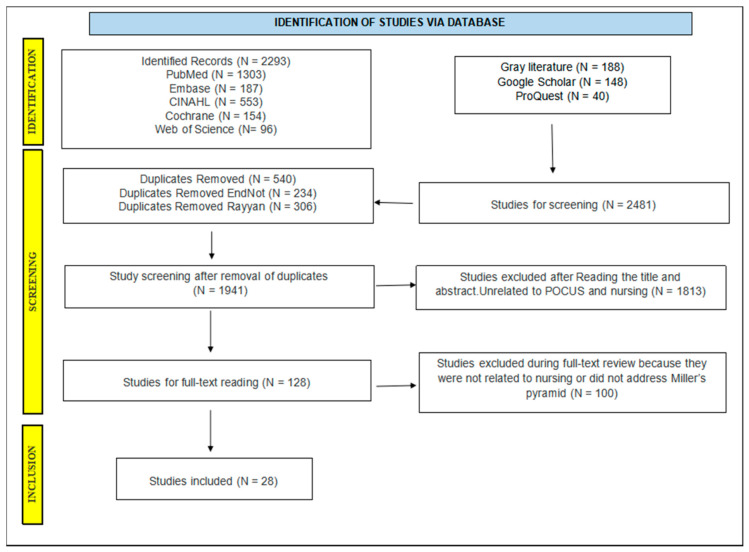
PRISMA-ScR flow diagram of study selection for the scoping review.

The search strategy identified 2481 records, including 2293 records from bibliographic databases and 188 from gray literature sources. After the removal of 540 duplicates, 1941 records remained for title and abstract screening. Of these, 1813 records were excluded because they did not meet the eligibility criteria. A total of 128 full-text articles were assessed for eligibility, and 100 were excluded for the following reasons: population not aligned with the review objective (e.g., studies focused exclusively on physicians or other health professionals without explicitly including nurses), ultrasound use unrelated to point-of-care applications, absence of competency-related outcomes or descriptions, and insufficient relevance to the review question. Twenty-eight studies met the inclusion criteria and were included in the final synthesis. The complete study selection process is presented in the PRISMA-ScR [17] flow diagram (Figure 1).

### 3.2. Characteristics of Included Studies

**Figure 2:**
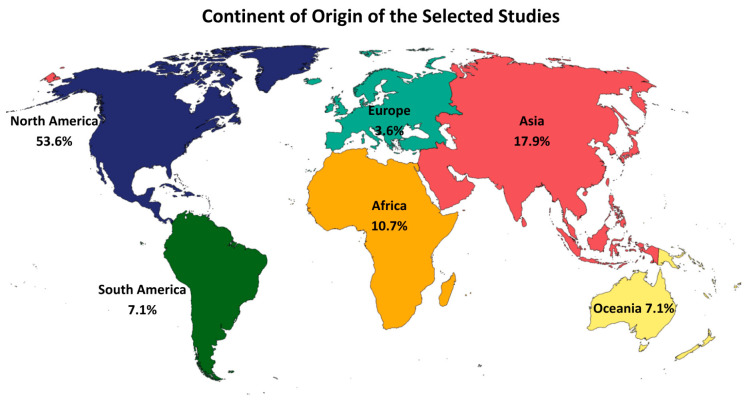
Geographic distribution of included studies by continent.

**Figure 3:**
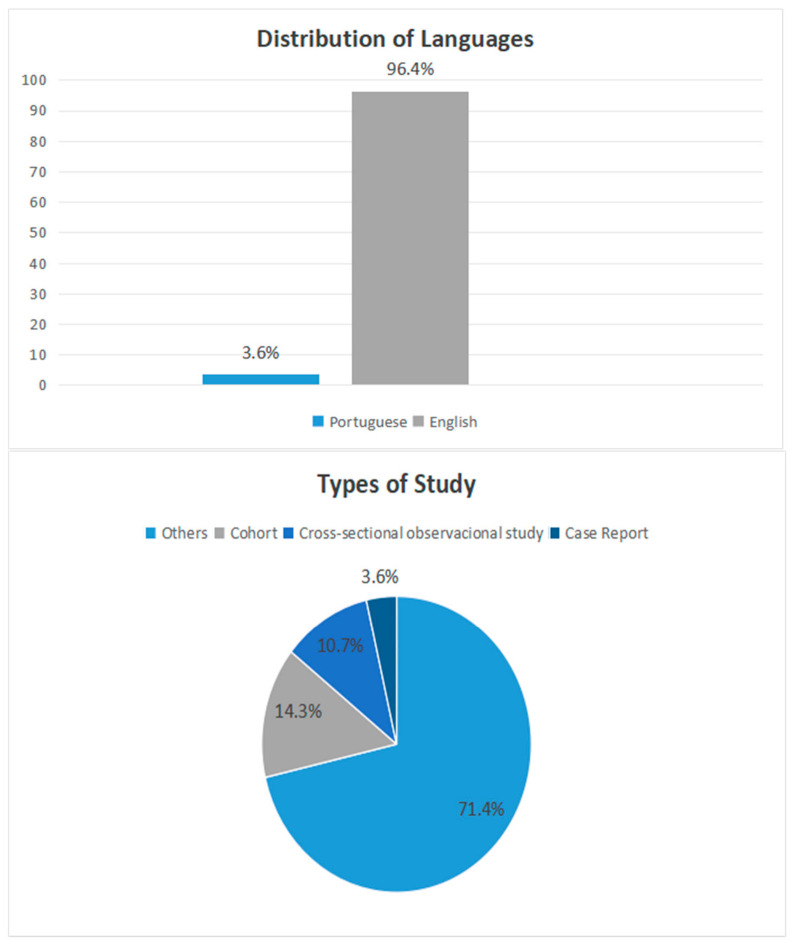
Distribution of included studies by publication language and study design.

The 28 included studies were published between 2013 and 2025, with a marked increase in publications over the past decade. A summary of the main characteristics of the included studies is provided in Table 1. Most studies originated from North America (53.6%) [19,20,21,22,23,24,25,26,27,28,29,30,31,32,33] followed by Asia (17.9%) [34,35,36,37,38] Africa (10.7%) [13,39,40], Oceania (7.1%) [41,42] South America (7.1%) [43,44] and Europe (3.6%) [45] (Figure 2). English was the predominant language of publication (96.4%) [13,19,20,21,22,23,24,25,26,27,28,29,30,31,32,33,34,35,36,37,38,39,40,41,42,44,45] (Figure 3).

A wide range of methodological designs was identified, including cohort studies [23,24,25,35], cross-sectional observational studies [20,27,44], prospective pre–post designs [13,36], quality improvement initiatives [26,28,29,41], exploratory studies [39], and case reports [21] (Figure 3). Some studies were grouped under the category “Other” because they represented a small number of methodologically specific or heterogeneous designs that did not justify further subdivision for descriptive synthesis. Clinical settings included emergency departments [21], intensive care units [28,29,44], medical–surgical wards [24,26,39], outpatient services, and educational or simulation-based environments [25,37]. Most studies focused on graduate nurses or nurse practitioners, who generally had no previous formal training, certification, or clinical exposure to PoCUS before participating in the educational interventions.

### 3.3. Mapping of Clinical Competencies According to Miller’s Pyramid

**Figure 4:**
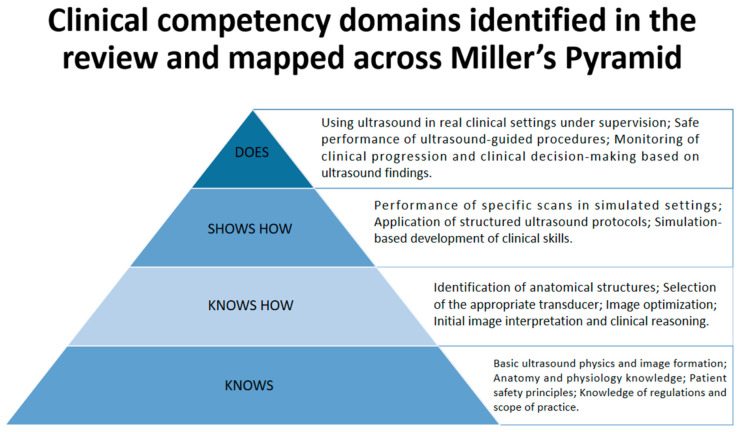
Clinical competencies for nursing use of point-of-care ultrasound mapped to Miller’s Pyramid.

Clinical competencies required for nurses to use point-of-care ultrasound were identified across all four hierarchical levels of Miller’s Pyramid [14]. Certain competencies were explicitly delineated by the original authors, whereas others were deduced through interpretive mapping based on the educational outcomes, procedural performance, assessment strategies, and clinical activities reported within the selected literature. However, the distribution of competencies was uneven across levels, with a marked predominance of competencies related to technical execution and procedural performance. Competencies involving interpretive judgment, clinical reasoning, and autonomous decision-making were less consistently described. Overall, the evidence suggests that nursing competence in point-of-care ultrasound is multidimensional and developmental, encompassing foundational knowledge, applied understanding, technical performance, and integration of ultrasound findings into clinical care. Figure 4 summarizes the main clinical competency domains identified in the review and maps them across the four levels of Miller’s Pyramid.

#### 3.3.1. Competencies at the “Knows” Level (Theoretical Knowledge)

At the foundational level, the literature described competencies related to the theoretical basis required for the safe and meaningful use of point-of-care ultrasound. These included understanding basic ultrasound physics and image formation [26,36,45], knowledge of anatomy and physiology relevant to the anatomical region examined or the clinical application [23,26,40,42], knowledge of patient safety principles [26], and knowledge of regulations and professional boundaries for point-of-care ultrasound use in nursing practice [39]. In addition, some studies expanded this foundational dimension by including theoretical knowledge related to vascular access and ultrasound-guided cannulation [19,20,28,31,44], basic echocardiographic concepts and standard views [40], the cricothyroid membrane and airway sonoanatomy [30], swallowing-related ultrasonographic assessment [36], fetal heart rate assessment using ultrasound [25], bladder ultrasonography and residual urine evaluation [35,43], and ultrasound-based tip location during placement of central venous access devices [38]. Theoretical knowledge was also incorporated into educational approaches focused on obstetric ultrasound [13,39], advanced nursing health assessment [22], and structured simulation-based learning for nurse practitioners and nursing students [35,37].

Although this level was widely represented, theoretical knowledge was often described only briefly, and many studies did not clearly articulate whether it was linked to explicit learning objectives, validated cognitive assessments, or progression to higher levels of competence. In several cases, knowledge was treated as a prerequisite for subsequent technical training rather than as a competency domain requiring structured evaluation in its own right.

#### 3.3.2. Competencies at the “Knows How” Level (Application and Clinical Reasoning)

Competencies at the “knows how” level involved applying theoretical knowledge to practical and clinical situations, particularly in ways requiring judgment, adaptation, and interpretation. These included differentiation of anatomical structures [29], selection of the appropriate transducer according to examination type and patient presentation [23,40,45], optimization of image quality through adjustment of gain, focus, depth, and related parameters [29,45], and initial image interpretation and clinical reasoning [45]. This level also encompassed recognition of vascular anatomy and selection of the most appropriate vessel or puncture site for cannulation [20,24,26,31,41,44]; accurate identification of tip location during placement of central venous access devices [38], identification of fetal cardiac activity and determination of fetal heart rate [25], interpretation of findings related to aspiration and pharyngeal post-swallow residue [36], interpretation of bladder ultrasound findings to support clinical decision-making [35,43], and understanding of cardiovascular, pulmonary, abdominal, and lower extremity venous ultrasound findings in educational settings [22,37].

This level was inconsistently described across the included studies. Although many training programs taught nurses how to acquire images, fewer explicitly addressed how nurses should interpret those images, recognize uncertainty, or integrate ultrasound findings into nursing reasoning and clinical judgment. As a result, the “knows how” level appeared less robustly operationalized than the levels focused on knowledge or technical performance.

#### 3.3.3. Competencies at the “Shows How” Level (Demonstrated Performance)

At the “shows how” level, studies emphasized the demonstration of technical and procedural competence in controlled, educational, or simulated settings. Common competencies included demonstration of ultrasound-related clinical skills through examinations, tests, or simulated scenarios [42,45], performance of specific scans in simulated environments, such as vascular, pulmonary, abdominal, bladder, cardiac, and lower extremity venous scans [22,34,35,37,42], and application of structured ultrasound protocols, including trauma and rapid assessment protocols such as focused assessment with sonography for trauma, extended focused assessment with sonography for trauma, and rapid ultrasound in shock protocols [13,41]. Demonstrated performance also included simulation-based practice of ultrasound-guided peripheral intravenous catheter placement [19,20,24,29,31,44], simulation-based or cadaver-based performance of needle cricothyrotomy under ultrasound guidance [30], simulation-based determination of fetal heart rate [25], and objective structured clinical examination for swallowing-related ultrasound monitoring [36].

Some studies also incorporated structured simulation exercises, hands-on practice, direct observation, and practical checklists to document learners’ ability to operate the device, identify structures, acquire interpretable images, and perform ultrasound-guided procedures [20,24,29,36,42]. In nursing education contexts, this level included the use of ultrasound to enhance cardiac, pulmonary, and abdominal assessment skills, enabling students to demonstrate performance while linking ultrasound findings to physical examination [22]. In studies involving nurse practitioners and nursing students, demonstrated performance also included image acquisition and interpretation testing immediately after the course and at follow-up [35,37], as well as the development of clinical skills through gamified learning approaches [32].

Simulation-based training and supervised practical exercises were the most common strategies used to support this level of competence. Although the “shows how” level was among the most frequently represented levels in the included literature, assessment methods varied substantially across studies, and explicit performance criteria, benchmark standards, and validated evaluation tools were seldom reported consistently.

#### 3.3.4. Competencies at the “Does” Level (Autonomous Clinical Practice)

Competencies at the apex of the pyramid related to the use of point-of-care ultrasound in real clinical environments, particularly when nurses used ultrasound to support care decisions, monitor progression, or perform procedures in practice settings. These included using point-of-care ultrasound in real clinical environments with patients under supervision [42], safe performance of ultrasound-guided procedures within nursing scope of practice, especially vascular access and venous assessment [20,21,24,26,27,34,44], and monitoring clinical progression and making decisions based on ultrasound findings [23,26,45]. This level also included clinical use of bladder ultrasound to support the assessment of urinary retention and guide therapeutic conduct [35,43], use of ultrasound in critical care transport to support bedside assessment and management decisions [23], use of ultrasound in hemodialysis settings to assess arteriovenous access and guide cannula placement [41], and use of ultrasound in real-world peripheral intravenous access programs or training pathways that translated into bedside performance [21,26,29,31].

Some studies also suggested movement toward autonomous practice by documenting increased use of ultrasound examinations in daily practice after training [13,37], clinical implementation of ultrasound-guided vascular access by nurses previously trained through simulation and supervised insertion [29,31], and improvement in procedural success in actual clinical care [28]. In swallowing care, the “does” level was suggested when nurses used ultrasound-derived findings to guide aspiration-related care decisions, although real-world autonomous application was less fully documented than performance in structured assessments [36]. Similarly, in fetal heart rate assessment, competency achievement was linked to supervised bedside application in pregnant patients in the emergency department [25].

Fewer studies addressed this level explicitly than the lower levels of the pyramid. When reported, autonomous practice was often inferred from clinical implementation, frequency of use, or procedural success rather than formally and longitudinally assessed through validated frameworks. As a result, the literature remains limited in its ability to demonstrate whether training programs truly support sustained progression from supervised performance to consistent, context-sensitive, and autonomous nursing use of point-of-care ultrasound in everyday practice.

## 4. Discussion

This scoping review demonstrates that, although the use of point-of-care ultrasound by nurses is expanding globally, the development of clinical competence for PoCUS remains conceptually fragmented and inconsistently supported by current educational and assessment approaches [3,37,46]. The competencies identified in the included studies can be broadly grouped into four domains: foundational knowledge of ultrasound principles, anatomy, physiology, and professional regulations; application competencies involving image optimization, structure recognition, and interpretation of findings; technical and procedural competencies related to image acquisition and ultrasound-guided interventions; and competencies associated with integration of PoCUS findings into clinical decision-making and patient management.

However, these domains were unevenly represented across the literature. Most studies emphasized competencies related to technical execution and procedural performance, particularly image acquisition and ultrasound-guided procedures [20,21,42], whereas competencies involving image interpretation, clinical reasoning, diagnostic judgment, and autonomous decision-making were described less consistently. The application of Miller’s Pyramid [14] as an analytical framework revealed important discontinuities in the progression from knowledge acquisition to independent clinical practice, highlighting the need for nursing-specific competency frameworks capable of aligning curriculum design, assessment strategies, and scope-of-practice requirements with safe and effective PoCUS implementation [37].

This imbalance has direct implications for patient safety. Without explicit training and assessment in interpretive and decision-making competencies, nurses may be expected to use PoCUS in practice without sufficient preparation to manage equivocal findings, recognize the limits of the examination, or escalate care appropriately. These gaps are particularly concerning in high-acuity settings, where PoCUS findings may directly inform time-sensitive clinical interventions [21,28,29,44].

An alternative explanation for the predominance of technical competencies is the nature of the available literature itself. Many educational interventions and training programs prioritize outcomes that are easier to measure objectively, such as image acquisition, procedural success, and technical skill performance [20,37,45]. By contrast, competencies related to clinical reasoning, interpretation, recognition of uncertainty, and contextual decision-making are inherently more difficult to operationalize and assess consistently [45,47]. Consequently, the current distribution of competencies may reflect not only educational priorities in PoCUS training but also methodological tendencies within published studies.

From an educational standpoint, the findings suggest that PoCUS training for nurses is frequently organized around short-term, skill-based interventions rather than longitudinal competency-based curricula. Although simulation-based education and supervised practice are widely reported [22,25,29,37], these strategies are often implemented without clearly defined learning outcomes linked to progressive levels of competence. A longitudinal competency-based model could support progression from theoretical foundations (“knows”), to supervised application and interpretation (“knows how”), demonstration of competence in simulation or structured assessments (“shows how”), and ultimately workplace-based performance in real clinical settings (“does”). Such an approach may better support competency acquisition, retention, and readiness for autonomous clinical practice [37].

Competency-based education frameworks emphasize alignment among learning objectives, teaching strategies, and assessment methods across successive stages of professional development [29,37,48]. In this review, the application of Miller’s Pyramid [14] underscores the importance of designing PoCUS curricula that deliberately support progression from foundational knowledge to independent clinical performance. For nursing education, this implies moving beyond procedural instruction toward integrated learning experiences that foster clinical reasoning, reflective practice, and contextual judgment [19]. Such an approach is especially relevant in undergraduate and graduate nursing programs, where PoCUS is increasingly being introduced, yet often without standardized expectations regarding competence [19,22,37].

A central gap identified in this review is the limited availability of nursing-specific tools for assessing PoCUS competence. Although some studies described practical checklists, direct observation methods, simulation-based evaluations, objective structured clinical examinations, and motion-analysis approaches [19,22,24,25,29,36,37,42,45], assessment strategies remained highly heterogeneous and were rarely grounded in validated competency frameworks [20,44]. These approaches represent promising foundations for future competency assessment frameworks; however, none has yet emerged as a fully validated nursing-specific instrument, and further studies are needed to establish their reliability, validity, and applicability across diverse nursing practice contexts, thereby enabling more robust evaluation of PoCUS competence in clinical practice [20].

These findings also have important implications for regulatory and professional governance. Internationally, the PoCUS implementation by nurses occurs across diverse healthcare contexts and professional roles, ranging from procedural applications such as vascular access to broader assessment and monitoring activities [3,49,50,51,52]. Such variability suggests that competency expectations may differ according to local educational, professional, and regulatory frameworks.

In Brazil, for example, nurses are permitted to use PoCUS within defined boundaries, with regulations emphasizing technical image acquisition and restricting diagnostic interpretation [10]. However, in the absence of clearly defined nursing competencies, the practical operationalization of these regulations remains challenging. Nurses may be authorized to perform PoCUS without explicit guidance regarding the degree of interpretive judgment expected or the competencies required to support safe clinical decision-making. Consequently, competency frameworks capable of aligning educational preparation, assessment strategies, and regulatory expectations may contribute to safer and more consistent PoCUS implementation across healthcare settings [3,37].

From an ethical perspective, expanding PoCUS use without proportional investment in competency development and assessment raises concerns related to professional accountability and patient safety. Clear, nursing-specific competency frameworks are therefore essential to ensure that PoCUS is incorporated into practice in a manner consistent with the ethical principles of beneficence, non-maleficence, and professional responsibility.

This review also identifies several priorities for future research. Studies are needed that move beyond descriptive accounts of training programs and examine the development, assessment, and retention of PoCUS competencies over time. Longitudinal studies, validation studies of assessment instruments, and investigations examining associations between nursing PoCUS competence and patient outcomes are particularly warranted. In addition, evidence from low- and middle-income countries remains limited, underscoring the need for context-sensitive research that considers resource constraints and variability in nursing scope of practice.

Developing a validated, nursing-specific instrument for assessing PoCUS competence, anchored in Miller’s Pyramid [14], represents a critical next step. Such an instrument could support educational planning, inform regulatory decision-making, and contribute substantially to the safe and effective integration of PoCUS into nursing practice.

### Limitations of This Review

This scoping review has some limitations that should be considered when interpreting the findings. Crucially, although a comprehensive search was conducted across multiple bibliographic databases and gray literature sources, some relevant publications may not have been identified, particularly unpublished materials or documents not indexed in the selected platforms. Furthermore, the included literature was methodologically heterogeneous and often employed differing terminology to describe competence, skills, proficiency, and assessment, which may have limited the consistency of data charting and synthesis. In this regard, because many primary studies did not explicitly define competence as a theoretical construct, certain competency domains were identified via interpretive mapping of educational outcomes, procedural performance, assessments, and clinical practices. While this process was executed systematically and independently by two reviewers, a degree of subjectivity remains an inherent limitation.

In addition, the relatively small number of included studies appears to reflect both the emerging nature of nursing PoCUS research and the limited number of publications that explicitly address competency development or assessment. Many studies involving nurse-performed ultrasound focus primarily on clinical outcomes, procedural implementation, or educational interventions without providing sufficient detail regarding competency definitions or assessment processes to meet the inclusion criteria of this review.

Notably, in accordance with methodological recommendations for scoping reviews, no formal critical appraisal of methodological quality was undertaken. Consequently, the findings should be interpreted as a mapping of the available evidence rather than as an evaluation of the robustness or effectiveness of the included studies. Additionally, several sources provided limited detail regarding competency definitions, assessment methods, or the level of nursing autonomy in practice, which may have constrained the precision of mapping to Miller’s Pyramid. Lastly, because the synthesis was oriented toward nursing-specific competencies, some potentially informative evidence from the broader multiprofessional ultrasound literature may not have been fully captured unless nurses were explicitly included. Despite these limitations, this review offers a structured and theory-informed synthesis of the available evidence and identifies important gaps to guide future research, education, and professional regulation.

## 5. Conclusions

This scoping review synthesized global evidence on the clinical competencies required for nurses to use point-of-care ultrasound and demonstrated that current approaches to competency development remain uneven, with a predominant emphasis on technical skill acquisition. When examined through the lens of Miller’s Pyramid, important areas requiring further investigation became apparent in the progression from foundational knowledge to autonomous clinical practice, particularly in competencies involving clinical reasoning, interpretive judgment, and decision-making in real care settings.

These findings underscore the need for nursing-specific competency frameworks that move beyond procedural performance and support longitudinal, competency-based education aligned with professional scope of practice, ethical responsibilities, and patient safety requirements. The limited availability of standardized and validated assessment strategies further highlights the urgency of developing instruments capable of evaluating competence across the full spectrum of clinical performance.

By mapping nursing PoCUS competencies within a structured theoretical framework, this review provides a foundation for advancing educational programs, assessment methods, and regulatory guidance. Strengthening competency development in this way is essential not only to improve training quality, but also to ensure that point-of-care ultrasound is integrated into nursing practice in a safe, ethical, context-sensitive, and clinically meaningful manner across diverse healthcare settings.

## Figures and Tables

**Table 1:** Summary of Characteristics of the Included Studies.

Author (Year)	Country	Setting	Type of Study	Competency Focus
Kaganovskaya (2021) [19]	United States of America	Not informed	Other/Educational program development study	Knowledge of ultrasound-guided vascular access; simulation-based development of skills for ultrasound-guided peripheral intravenous catheter placement.
Wanjiku (2018) [13]	Kenya	Other	Other/Prospective pre–post study	Application of structured ultrasound protocols; performance of obstetric, thoracic, cardiac, and trauma-related ultrasound examinations in supervised training settings.
Adhikari (2015) [20]	United States of America	Emergency department	Cross-sectional observational study	Basic ultrasound principles; identification of vascular anatomy; vein selection for cannulation; safe performance of ultrasound-guided vascular access.
Moore (2013) [21]	United States of America	Emergency department	Case report	Safe performance of ultrasound-guided peripheral intravenous access; selection of appropriate venous access sites; structured training for difficult vascular access.
Mubuuke (2023) [39]	Uganda	Medical clinic	Other/Exploratory qualitative study	Knowledge of regulations and scope of practice; obstetric ultrasound assessment, including fetal identification, fetal heartbeat, fetal presentation, placental location, cervical length, amniotic fluid, fetal age estimation, professionalism, and ethics.
Abe-Doi (2024) [34]	Japan	Other	Other	Selection of puncture points using ultrasound support.
Engelman (2017) [40]	Uganda	Other	Other/Prospective single-group pre–post study	Anatomy and physiology knowledge; transducer selection; knowledge of standard echocardiographic views; image optimization; image interpretation in echocardiography training.
Silva (2023) [43]	Brazil	Intensive Care Unit	Other/Integrative review	Bedside ultrasound assessment of urinary retention; image interpretation; clinical decision-making; use in real clinical settings.
Hauglum (2022) [22]	United States of America	Other	Other/Educational innovation report	Cardiac, pulmonary, and abdominal image acquisition and interpretation; anatomy-based learning; ultrasound-supported physical assessment skills.
Cover (2019) [23]	United States of America	Other	Cohort study	Anatomy and physiology knowledge; transducer selection; monitoring of clinical progression; clinical decision-making based on ultrasound findings in critical care transport.
Good (2019) [24]	United States of America	Medical Clinic	Cohort study	Ultrasound-guided peripheral intravenous catheter placement; identification of veins using ultrasound; objective assessment of procedural competence.
Miles (2023) [25]	United States of America	Emergency Department	Cohort study	Determination of fetal heart rate using bedside ultrasound; performance in simulated settings; supervised achievement of competency in clinical practice.
Naito (2025) [35]	Japan	Not informed	Cohort study	Bladder ultrasound image acquisition; image optimization; systematic examination; image interpretation; documentation of examination; clinical decision-making.
Oliveira (2016) [44]	Brazil	Intensive Care Unit	Cross-sectional observational study	Basic ultrasound physics and image formation; theoretical knowledge for ultrasound-guided peripheral venipuncture; safe performance of ultrasound-guided peripheral venipuncture; support for clinical decision-making.
Reeves (2017) [26]	United States of America	Medical Clinic	Other/quality improvement initiative	Basic ultrasound physics and image formation; anatomy and physiology knowledge; patient safety principles; safe performance of ultrasound-guided peripheral intravenous catheter insertion; monitoring of clinical progression and decision-making based on ultrasound findings.
Erickson (2014) [27]	United States of America	Emergency Department	Cross-sectional observational study	Basic ultrasound principles; identification of anatomical structures; ultrasound-guided small-vessel cannulation; safe procedural performance.
Pitman (2023) [28]	United States of America	Other	Other/quality improvement project	Ultrasound-guided vascular access knowledge; hands-on procedural competence; safe placement of peripheral intravenous, arterial, and central venous catheters with ultrasound assistance.
Hill (2024) [41]	Australia	Other	Other/quality improvement initiative	Arteriovenous access assessment with point-of-care ultrasound; visualization of cannula placement; identification of suitable areas for cannulation; recognition of vascular structures and cannulation-related complications.
Hackett (2021) [29]	United States of America	Intensive Care Unit	Other/quality improvement initiative	Identification of anatomical structures; image optimization; ultrasound-guided peripheral intravenous access; site selection and procedural preparation.
Garrett (2023) [30]	United States of America	Other	Other/quality improvement project	Ultrasound-guided needle cricothyrotomy; identification of the cricothyroid membrane; knowledge of ultrasound principles and equipment; procedural performance.
Yoshida (2020) [36]	Japan	Other	Other/prospective descriptive study	Monitoring of aspiration and pharyngeal post-swallow residue using ultrasound; acquisition and maintenance of adequate pharyngeal images; image interpretation for swallowing care.
Tolsgaard (2013) [45]	Denmark	Not informed	Other/Delphi consensus survey	Basic ultrasound principles; transducer selection; image optimization; initial image interpretation; clinical reasoning; documentation of examination; clinical decision-making.
Steinwandel (2018) [42]	Australia	Not informed	Other/Feasibility study	Anatomy and physiology knowledge; image acquisition; image interpretation; performance of inferior vena cava ultrasound assessment in simulated and clinical settings.
Filipovich (2021) [31]	United States of America	Outpatient	Other/Educational intervention study	Knowledge and confidence in ultrasound-guided peripheral intravenous catheter insertion; ultrasound use for peripheral access in critical situations.
Yamada (2023) [37]	Japan	Not informed	Other	Image acquisition; image interpretation; performance of cardiac, lung, lower extremity venous, and abdominal ultrasound.
Haamankuli (2025) [32]	United States of America	Other	Other/Educational innovation article	Knowledge and comfort using ultrasound; clinical skills.
Schott (2025) [33]	United States of America	Not informed	Other/Narrative review	Critical care ultrasound competence across cognitive, psychomotor, and affective domains; image acquisition; image interpretation; thoracic, vascular, abdominal, and cardiac ultrasound; ultrasound-guided bedside procedures.
Shen (2025) [38]	China	Other	Othe/Educational program development study	Basic knowledge of ultrasound-based tip location; accurate identification of tip location during placement of central venous access devices.

## Data Availability

No new data were created or analyzed in this study. Data sharing is not applicable to this article.

## Abbreviations

The following abbreviations are used in this manuscript:

POCUS	Point-of-care ultrasound

## Appendix A

### Appendix A.1. PubMed (National Institutes of Health) Search Strategies (–Last Run: 9 April 2025)

Search Strategy for Pubmed	
Search 1	nurse* OR nursing* OR staff* OR team* OR personnel*
Search 2	(“point-of-care” OR bedside* OR “bed-side” OR POC OR ultrasound* OR ultrasonograph* OR Echotomograph* OR Sonograph* OR Echograph* OR Echocardiograph* OR Echoencephalograph* OR Endosonograph* OR Echo-Endoscop* OR Ultrasonic OR POCUS OR Diagnostic Ultrasounds OR “Ultrasound Imaging” OR “Bedside Test” OR “Bedside Testing” OR “Bedside Technolog*” OR “Point of Care Technology” OR “Point of Care Testing” OR “Point-of-care ultrasonography” OR “Point-Of-Care Ultrasound”)
Search 3	“Clinical Competenc*” OR “Clinical Skil*”
Search 4	1 AND 2 AND 3
Note: The asterisk indicates (*) truncation (wildcard) and was used to retrieve terms sharing the same word root but having different endings, thereby increasing the sensitivity and comprehensiveness of the search.	

### Appendix A.2. Embase (Elsevier) Search Strategies (–Last Run: 9 April 2025)

Search Strategy for Embase	
Search 1	nurse* OR nursing* OR staff* OR team* OR personnel*
Search 2	(“point-of-care” OR bedside* OR “bed-side” OR POC OR ultrasound* OR ultrasonograph* OR Echotomograph* OR Sonograph* OR Echograph* OR Echocardiograph* OR Echoencephalograph* OR Endosonograph* OR Echo-Endoscop* OR Ultrasonic OR POCUS OR Diagnostic Ultrasounds OR “Ultrasound Imaging” OR “Bedside Test” OR “Bedside Testing” OR “Bedside Technolog*” OR “Point of Care Technology” OR “Point of Care Testing” OR “Point-of-care ultrasonography” OR “Point-Of-Care Ultrasound”)
Search 3	“Clinical Competenc*” OR “Clinical Skil*”
Search 4	1 AND 2 AND 3
Note: The asterisk indicates (*) truncation (wildcard) and was used to retrieve terms sharing the same word root but having different endings, thereby increasing the sensitivity and comprehensiveness of the search.	

### Appendix A.3. CINAHL (EBSCOhost) Search Strategies (–Last Run: 9 April 2025)

Search Strategy for CINAHL	
Search 1	nurse* OR nursing* OR staff* OR team* OR personnel*
Search 2	(“point-of-care” OR bedside* OR “bed-side” OR POC OR ultrasound* OR ultrasonograph* OR Echotomograph* OR Sonograph* OR Echograph* OR Echocardiograph* OR Echoencephalograph* OR Endosonograph* OR Echo-Endoscop* OR Ultrasonic OR POCUS OR Diagnostic Ultrasounds OR “Ultrasound Imaging” OR “Bedside Test” OR “Bedside Testing” OR “Bedside Technolog*” OR “Point of Care Technology” OR “Point of Care Testing” OR “Point-of-care ultrasonography” OR “Point-Of-Care Ultrasound”)
Search 3	“Clinical Competenc*” OR “Clinical Skil*”
Search 4	1 AND 2 AND 3
Note: The asterisk indicates (*) truncation (wildcard) and was used to retrieve terms sharing the same word root but having different endings, thereby increasing the sensitivity and comprehensiveness of the search.	

### Appendix A.4. Cochrane Search Strategies (–Last Run: 9 April 2025)

Search Strategy for Cochrane	
Search 1	nurse* OR nursing* OR staff* OR team* OR personnel*
Search 2	(“point-of-care” OR bedside* OR “bed-side” OR POC OR ultrasound* OR ultrasonograph* OR Echotomograph* OR Sonograph* OR Echograph* OR Echocardiograph* OR Echoencephalograph* OR Endosonograph* OR Echo-Endoscop* OR Ultrasonic OR POCUS OR Diagnostic Ultrasounds OR “Ultrasound Imaging” OR “Bedside Test” OR “Bedside Testing” OR “Bedside Technolog*” OR “Point of Care Technology” OR “Point of Care Testing” OR “Point-of-care ultrasonography” OR “Point-Of-Care Ultrasound”)
Search 3	“Clinical Competence” OR “Clinical Competencies” OR “Clinical Skill” OR “Clinical Skills”
Search 4	1 AND 2 AND 3
Note: The asterisk indicates (*) truncation (wildcard) and was used to retrieve terms sharing the same word root but having different endings, thereby increasing the sensitivity and comprehensiveness of the search.	

### Appendix A.5. Web of Science Core Collection (Clarivate) Search Strategies (–Last Run: 9 April 2025)

Search Strategy for Web of Science Core Collection	
Search 1	nurse* OR nursing* OR staff* OR team* OR personnel*
Search 2	(“point-of-care” OR bedside* OR “bed-side” OR POC OR ultrasound* OR ultrasonograph* OR Echotomograph* OR Sonograph* OR Echograph* OR Echocardiograph* OR Echoencephalograph* OR Endosonograph* OR Echo-Endoscop* OR Ultrasonic OR POCUS OR Diagnostic Ultrasounds OR “Ultrasound Imaging” OR “Bedside Test” OR “Bedside Testing” OR “Bedside Technolog*” OR “Point of Care Technology” OR “Point of Care Testing” OR “Point-of-care ultrasonography” OR “Point-Of-Care Ultrasound”)
Search 3	“Clinical Competenc*” OR “Clinical Skil*”
Search 4	1 AND 2 AND 3
Note: The asterisk indicates (*) truncation (wildcard) and was used to retrieve terms sharing the same word root but having different endings, thereby increasing the sensitivity and comprehensiveness of the search.	

### Appendix A.6. Google Scholar Search Strategies (–Last Run: 9 April 2025)

Search Strategy for Google Scholar	
Search 1	“Point of care ultrasound” AND “Clinical competence” AND “Nurse”

### Appendix A.7. ProQuest Dissertations & Theses Global Search Strategies (–Last Run: 9 April 2025)

Search Strategy for ProQuest Dissertations & Theses Global	
Search 1	“Point of care ultrasonography” AND “Competence” AND “Nursing”

## References

[1] Justo G., Outuki M. (2024). Care protocols with POCUS. Rev. Socesp.

[2] Itoh T. (2020). Point-of-care ultrasound for pediatric endotracheal tube placement confirmation by advanced practice transport nurses. Air Med. J..

[3] Totenhofer R., Luck L., Wilkes L. (2021). Point-of-care ultrasound use by registered nurses and nurse practitioners in clinical practice: An integrative review. Collegian.

[4] Gimenes F.R.E., Rigobello M.C.G., Motta A.P.G., Pereira R.A., Napoleão A.A., Lopes C.T., Silva V.M. (2019). The nursing process in the management of risks associated with healthcare. PRONANDA Programa de Atualização em Diagnósticos de Enfermagem: Ciclo 7.

[5] Prado P.R., Zamarioli C.M., Gimenes F.R.E., NANDA International Inc. (2023). Application of the Outcome-Present State Test clinical reasoning model in critical patient care. Coletânea Diagnósticos de Enfermagem: Ciclo 1.

[6] Pontet J. (2019). Impact of an ultrasound-driven diagnostic protocol at early intensive-care stay: A randomized-controlled trial. Ultrasound J..

[7] Ünlüer E.E., Karagöz A., Oyar O., Vandenberk N., Kiyançiçek S., Budak F. (2014). Lung ultrasound by emergency nursing as an aid for rapid triage of dyspneic patients: A pilot study. Int. Emerg. Nurs..

[8] Schoch M. (2022). Point-of-care ultrasound-guided cannulation versus standard cannulation in hemodialysis vascular access: A controlled random order crossover pilot feasibility study. J. Vasc. Access.

[9] Hrics P., Wilber S., Blanda M.P., Gallo U. (1998). Ultrasound-assisted internal jugular vein catheterization in the emergency department. Am. J. Emerg. Med..

[10] Conselho Federal de Enfermagem (COFEN) Resolução COFEN No. 679/2021: Aprova a Normatização da Realização de Ultrassonografia à Beira do Leito e no Ambiente Pré-Hospitalar por Enfermeiro. https://www.cofen.gov.br/resolucao-cofen-no-679-2021/.

[11] Migot B.M., Passos M.V., Simões A.S.B., Marques A.L.M., Teodoro R.M.O., Faria E.C. (2024). Uso do ultrassom point-of-care (POCUS) por enfermeiros: Uma revisão integrativa. Rev. FT.

[12] Galon E.C., Ribeiro D.F.S., Terassi M. (2025). The usability of bedside ultrasound in nursing practice for critically ill patients. Rev. Bras. Enferm..

[13] Wanjiku G.W., Bell G., Wachira B.W. (2018). Assessing a novel point-of-care ultrasound training program for rural healthcare providers in Kenya. BMC Health Serv. Res..

[14] Miller G.E. (1990). The assessment of clinical skills/competence/performance. Acad. Med..

[15] Panúncio-Pinto M.P., Troncon L.E.A. (2014). Avaliação do estudante–aspectos gerais. Medicina.

[16] Peters M.D., Godfrey C., McInerney P., Munn Z., Tricco A.C., Khalil H., Aromataris E., Lockwood C., Porritt K., Pilla B., Jordan Z. (2024). Scoping Reviews. JBI Manual for Evidence Synthesis.

[17] Tricco A.C., Lillie E., Zarin W., O’Brien K.K., Colquhoun H., Levac D., Moher D., Peters M.D., Horsley T., Weeks L. (2018). PRISMA extension for scoping reviews (PRISMA-ScR): Checklist and explanation. Ann. Intern. Med..

[18] Open Science Framework. OSF: Open Science Framework. https://osf.io/dashboard.

[19] Kaganovskaya M., Wuerz L. (2021). Development of an educational program using ultrasonography in vascular access for nurse practitioner students. Br. J. Nurs..

[20] Adhikari S., Schmier C., Marx J. (2015). Focused simulation training: Emergency department nurses’ confidence and comfort level in performing ultrasound-guided vascular access. J. Vasc. Access.

[21] Moore C. (2013). An emergency department nurse-driven ultrasound-guided peripheral intravenous line program. J. Assoc. Vasc. Access.

[22] Hauglum A., Larrieu-Jimenez P. (2022). An innovative ultrasound-guided approach: Stimulating student query in advanced nursing health assessment. AANA J..

[23] Cover M., Tafoya C., Long B., Cranford J., Burkhardt J., Huang R., Theyyunni N., Bassin B., Lowell M., Kessler R. (2019). Creation of a flight nurse critical care ultrasound program. Air Med. J..

[24] Good R.J., Rothman K.K., Ackil D.J., Kim J.S., Orsborn J., Kendall J.L. (2019). Hand motion analysis for assessment of nursing competence in ultrasound-guided peripheral intravenous catheter placement. J. Vasc. Access.

[25] Miles G., Newcomb P., Spear D. (2023). Feasibility of teaching emergency department nurses to determine fetal heart rates using bedside ultrasound versus the hand-held Doppler. J. Radiol. Nurs..

[26] Reeves T., Morrison D., Altmiller G. (2017). A nurse-led ultrasound-enhanced vascular access preservation program: A quality improvement initiative combines advanced technology and patient-centered care. Am. J. Nurs..

[27] Erickson C.S., Liao M.M., Haukoos J.S., Douglass E., DiGeronimo M., Christensen E., Hopkins E., Bender B., Kendall J.L. (2014). Ultrasound-guided small vessel cannulation: Long-axis approach is equivalent to short-axis in novice sonographers experienced with landmark-based cannulation. West. J. Emerg. Med..

[28] Pitman J.S., Buscemi M., Funk E.M., Weaver S., Thompson J.A., Falyar C. (2023). Incorporating evidence-based ultrasound-guided vascular access standards into the nurse anesthetist armamentarium: A quality improvement project. J. Perianesth. Nurs..

[29] Hackett A., Wells C., Zhang Z., Kero J., Soriano J., Rivera J., Brito A., Guiney J., Leibner E., Kohli-Seth R. (2021). Development of a peripheral intravenous education program for nurses caring for pediatric intensive care unit patients. J. Pediatr. Nurs..

[30] Garrett S.G., Simmons Muckler V.C., Schmitt D.O., Hartwell E.H., Thompson J.A., Falyar C.R. (2023). Improving anesthesia providers’ needle cricothyrotomy success with ultrasound guidance: A cadaveric quality improvement project. AANA J..

[31] Filipovich S.J., Dilgard J.W., Conrad S.P., Moore C.B., Hefley J.B. (2021). Training program for ultrasound-guided intravenous catheter insertion. Mil. Med..

[32] Haamankuli H., Kopf S., White T., Suttle R., Azuero A., Nguyen S. (2025). Let the ultrasound games begin: Developing knowledge and clinical skills with gamification. J. Nurs. Educ..

[33] Schott C.K., Hernandez A., Pradhan D. (2025). Competencies, certification, and credentialing in critical care ultrasound. Crit. Care Clin..

[34] Abe-Doi M., Murayama R., Takahashi T., Matsumoto M., Tamai N., Nakagami G., Sanada H. (2024). Effects of ultrasound with an automatic vessel detection system using artificial intelligence on the selection of puncture points among ultrasound-beginner clinical nurses. J. Vasc. Access.

[35] Naito T., Hashizumi A., Sakai M., Yamamura E., Iwase M., Yamada K., Watanabe M., Kimura N., Kato T., Fujimoto E. (2025). Sustained effects of bladder point-of-care ultrasound simulation exercise on nursing students: A prospective cohort study. BMC Med. Educ..

[36] Yoshida M., Miura Y., Yabunaka K., Sato N., Matsumoto M., Yamada M., Otaki J., Kagaya H., Kamakura Y., Saitoh E. (2020). Efficacy of an education program for nurses on the use of point-of-care ultrasound to monitor aspiration and pharyngeal post-swallow residue: A prospective descriptive study. Nurse Educ. Pract..

[37] Yamada T., Ehara J., Funakoshi H., Endo K., Kitano Y. (2023). Effectiveness of point-of-care ultrasound (PoCUS) simulation course and skills retention for Japanese nurse practitioners. BMC Nurs..

[38] Shen Y., Zhou X., Yu J., Chang J., Li X., Li X., Zhang H. (2023). Development of an educational program for ultrasound-based tip location during placement of PICC-port to improve the competence of specialized nurse. J. Vasc. Access.

[39] Mubuuke A.G., Erem G., Nassanga R., Kiguli-Malwadde E. (2023). Point-of-care obstetric ultrasound training for midwives and nurses: Implementation and experiences of trainees at a rural hospital in sub-Saharan Africa: A qualitative study. BMC Res. Notes.

[40] Engelman D., Okello E., Beaton A., Selnow G., Remenyi B., Watson C., Longenecker C.T., Sable C., Steer A.C. (2017). Evaluation of computer-based training for health workers in echocardiography for RHD. Glob. Heart.

[41] Hill K., Jaensch A., Childs J.T., McDonald S. (2024). Evaluation of point-of-care ultrasound (POCUS) training on arteriovenous access assessment and cannula placement for haemodialysis. J. Vasc. Access.

[42] Steinwandel U., Gibson N., Towell-Barnard M., Rippey J., Rosman J. (2018). Educating renal nurses—Inferior vena caval ultrasound for intravascular volume assessment. Ren. Soc. Australas. J..

[43] Silva I.C., Schneider C., Silva L.F., Moreira J.R. (2023). Avaliação de retenção urinária pelo enfermeiro orientada por ultrassonografia à beira do leito: Revisão integrativa. Rev. Enferm. Atual Derme.

[44] Oliveira A.M., Danski M.T.R., Pedrolo E. (2016). Inovação tecnológica para punção venosa periférica: Treino com ultrassom. Rev. Bras. Enferm..

[45] Tolsgaard M.G., Todsen T., Sorensen J.L., Ringsted C., Lorentzen T., Ottesen B., Tabor A. (2013). International multispecialty consensus on how to evaluate ultrasound competence: A Delphi consensus survey. PLoS ONE.

[46] El Miedany Y., Palmer D. (2012). Musculoskeletal ultrasound: Examining the joints. Br. J. Nurs..

[47] Gottlieb M., Duanmu Y. (2023). Beyond the numbers: Assessing competence in point-of-care ultrasound. Ann. Emerg. Med..

[48] Bowman A., Reid D., Harreveld R.B., Lawson C. (2021). Evaluation of student clinical performance after simulation training. Radiography.

[49] Lo H. (2022). Ambulatory use of handheld point-of-care ultrasound (HH-POCUS) in rural Brandenburg: A pilot study. Ultraschall Med..

[50] Corcoran E., Hopkins P., Fisher R., Wong A., Rose L. (2023). Intensive care nurse-led point-of-care ultrasound in the assessment and management of the critically ill COVID-19 patient: A single-centre case series. Nurs. Crit. Care.

[51] Lucenti E. (2023). Ultrasound applied to nursing in the Emergency Medical Service (EMS): A scoping review. Inferm. J..

[52] Varndell W., Topacio M., Hagness C., Lemon H., Tracy D. (2018). Nurse-performed focused ultrasound in the emergency department: A systematic review. Australas. Emerg. Care.

